# Evidence for Impaired Renin Activity in Postural Orthostatic Tachycardia Syndrome

**DOI:** 10.3390/jcm12144660

**Published:** 2023-07-13

**Authors:** Jasmina Medic Spahic, Ingrid Yao Mattisson, Viktor Hamrefors, Madeleine Johansson, Fabrizio Ricci, Jan Nilsson, Olle Melander, Richard Sutton, Artur Fedorowski

**Affiliations:** 1 Department of Clinical Sciences, Lund University, 214 28 Malmö, Sweden; jasmina.medic_spahic@med.lu.se (J.M.S.); madeleine.johansson@med.lu.se (M.J.); jan.nilsson@med.lu.se (J.N.); olle.melander@med.lu.se (O.M.); artur.fedorowski@ki.se (A.F.); 2 Department of Internal Medicine, Skåne University Hospital, 214 28 Malmö, Sweden; ingrid.yao_mattisson@med.lu.se; 3 Department of Cardiology, Skåne University Hospital, 214 28 Malmö, Sweden; viktor.hamrefors@med.lu.se; 4 Department of Neuroscience, Imaging and Clinical Sciences, “G.d’Annunzio” University of Chieti-Pescara, Via dei Vestini 33, 66100 Chieti, Italy; 5 Fondazione Villa Serena per la Ricerca, 65013 Città Sant’Angelo, Italy; 6 National Heart and Lung Institute, Imperial College, Hammersmith Hospital Campus Du Cane Road, London W12 0HS, UK; r.sutton@imperial.ac.uk; 7 Department of Cardiology, Karolinska University Hospital, Department of Medicine, Karolinska Institute, 171 77 Stockholm, Sweden

**Keywords:** postural orthostatic tachycardia syndrome (POTS), renin, aldosterone, angiotensin

## Abstract

Background: Postural orthostatic tachycardia syndrome (POTS) is a heterogeneous condition predominantly affecting autonomic control of the cardiovascular system. Its extensive symptom diversity implies multi-organ involvement that interacts in ways still requiring full exploration. Current understanding of POTS pathophysiology suggests alterations in the renin–angiotensin–aldosterone system as a possible contributing factor. Therefore, we investigated the relationship between the activity of the renin–angiotensin–aldosterone system and hemodynamic parameters in a cohort of POTS patients and controls recruited at a tertiary referral center. Methods: The case-control study included 46 patients with POTS (27 ± 9 years), and 48 healthy controls (30 ± 9 years) without orthostatic intolerance. Plasma renin activity, expressed as angiotensin I generation, and plasma aldosterone were measured by enzyme-linked immunosorbent assay and were correlated with hemodynamic parameters obtained during active standing tests. Results: Renin activity was significantly downregulated in POTS patients compared to healthy individuals (median, 3406 ng/mL vs. 9949 ng/mL, *p* < 0.001), whereas aldosterone concentration did not differ between POTS and healthy controls (median, 218 pmol/L vs. 218 pmol/L, *p* = 0.26). A significant inverse correlation between renin activity and supine and orthostatic blood pressure levels was observed in healthy individuals (*p* < 0.05 for all), but not in POTS patients. Conclusions: Renin activity, but not aldosterone concentration, is downregulated in patients with POTS. Moreover, renin activity in POTS is dissociated from supine and standing blood pressure levels in contrast to healthy individuals. These findings suggest impaired renin function in POTS, which may direct future therapeutic approaches.

## 1. Introduction 

Postural orthostatic tachycardia syndrome (POTS) is a dysautonomic disorder characterized by an excessive increase in heart rate when upright, accompanied by a broad spectrum of cardiovascular and non-cardiovascular symptoms [1,2,3]. POTS is a common cause of orthostatic intolerance predominantly found in younger females of childbearing years [4]. Current diagnostic criteria for POTS, outlined by the European Society of Cardiology Guidelines on Syncope, include symptoms of orthostatic intolerance for at least 3–6 months together with orthostatic heart rate (HR) increase >30 bpm (HR increase > 40 bpm in patients <19 years) or HR exceeding 120 bpm when upright in the absence of orthostatic hypotension [1,5,6]. The symptom variety experienced in POTS is very wide and involves several organs though the precise etiology is not fully understood. Cardiovascular changes are mandatory for the diagnosis but other constellations of symptoms like sleep disturbance, fatigue, and concentration difficulties are also extensively reported by POTS patients [7,8]. For this reason, different pathophysiological mechanisms have been suggested to interact and overlap in POTS, aiming to explain this complex clinical syndrome [4,9]. 

The renin–angiotensin–aldosterone system (RAAS) is a major neuroendocrine mechanism that regulates plasma sodium concentration, arterial blood pressure, and extracellular volume [10,11]. Interactions between RAAS and the autonomic nervous system have been previously established for cardiovascular regulation [12]. The regulatory mechanism involves input from renal, cardiovascular, and central nervous systems [11]. 

Previous studies have suggested that lower renin activity, lower estimated angiotensin-converting enzyme-2 activity [13] and lower aldosterone levels [14] observed in POTS may contribute to the dysregulation of sodium re-uptake, reduced blood volume, and impaired blood pressure control. Pathophysiological mechanisms underlying this phenomenon are unclear though it is believed that autoimmunity may play a crucial role through inappropriate activation of adrenergic and angiotensin receptors [15,16,17,18]. In this study, we aimed to explore renin activity and aldosterone concentration in POTS patients and healthy controls and to relate these results to resting and orthostatic hemodynamic parameters in both groups. 

## 2. Methods

### 2.1. Study Population

Patients were not involved in the design, conduct, reporting, or dissemination plans of our research. We included 46 patients with confirmed POTS recruited from a tertiary referral center at the Department of Cardiology, Skåne University Hospital in Malmö, Sweden, and 48 hospital staff members or volunteers that participated as controls. The controls had neither orthostatic intolerance symptoms nor history of syncope and were age-and-sex matched as closely as possible with POTS patients. Patients with POTS fulfilled predefined diagnostic POTS criteria, including symptoms of orthostatic intolerance for at least 6 months and a positive head-up tilt test (HUT), confirming heart rate (HR) increase > 30 beats per minute (bpm), or HR exceeding 120 bpm when tilted from supine to upright position, according to international consensus [2,19,20]. Prior to study initiation, it was ascertained that none of the participants had any known diagnosis of an autoimmune disorder. Additionally, there was a notable absence of prevalent history relating to atherosclerotic cardiovascular disease, hypertension, stroke, atrial fibrillation, or heart failure among the study cohort. These stringent inclusion criteria helped to ensure a participant pool free of these specified pre-existing conditions, enhancing the precision and relevance of our research findings.

### 2.2. Examination and Blood Sample Collection

All study participants underwent a standardized active standing test, as described below, at their blood sample collection visit to the Clinical Research Unit at Skåne University in Malmö between September 2017 and June 2019 [15]. Participants were asked to fast from 10 pm the night before and were instructed to discontinue cardiovascular medications 48 h before the visit. Both controls and POTS patients underwent an active standing test with monitoring of hemodynamic changes. Blood pressure (BP) was recorded using a non-invasive validated digital method (Boso Medicus, Bosch + Sohn GmbH und Co, KG, 72417 Jungingen, Germany) measured in the non-dominant arm twice when supine after 5 min rest and standing after 1, 3, and 5 min, after which the test was terminated. The mean values of blood pressure and heart rate measured at every time interval were calculated and used for statistical analyses. Only healthy individuals with normal active standing hemodynamic parameters and no symptoms of orthostatic intolerance during the test were included as controls. 

Blood samples were collected into EDTA vacutubes and centrifuged for 10 min at 2000× *g* within 4 h. Centrifuged samples were then poured into a new tube and kept for 15 h at room temperature and up to 2 days refrigeration. Frozen samples were transported to the laboratory. 

### 2.3. Determination of Renin Activity

**Figure 1:**
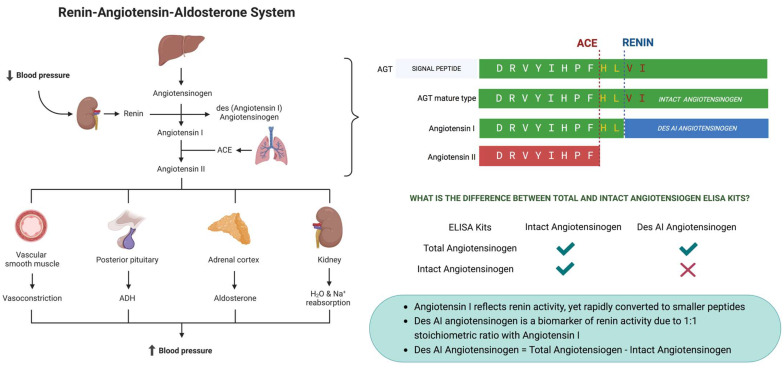
Renin–angiotensin–aldosterone system and assessment of renin activity.

EDTA plasma from POTS patients and controls was stored on ice. A plasma pool was created for normalization. Samples were diluted in three steps to 1:4000 (1:10, 1:100, and 1:4000). Enzyme-linked immunosorbent assay (ELISA) of human intact angiotensinogen and total angiotensinogen (Immuno-Biological Laboratories Co., Ltd., Fujioka-Shi, Gunma, Japan) was carried out according to the manufacturer’s instructions. Absorbance was measured with the Magellan instrument at OD450nm. Concentrations were obtained using the standard curve OD450nm, and a nonlinear quadratic curve fitted regression according to the manufacturer’s instructions. Angiotensin I generation was calculated as total angiotensinogen − intact angiotensinogen (Figure 1). Angiotensin I levels were interpreted as plasma renin activity (PRA) [21,22].

### 2.4. Determination of Aldosterone Concentration

The analytical method for determining aldosterone concentration in serum is based on extraction with supported liquid extraction technology, followed by online extraction and analysis with liquid chromatography-tandem mass spectrometry (LC-MS/MS). The LC-MS/MS system was equipped with a Nexera LC system (Shimadzu, Kyoto, Japan) and a Q-TRAP 5500 mass spectrometer (SCIEX, Framingham, MA, USA). Quantification of serum aldosterone concentration was performed using a deuterated internal standard. On the LC system, an additional purification step for the samples was first performed with an on-line extraction on a nitrile extraction column (Eclipse XDB CN 50 mm × 2.1 mm, 5 µm; Agilent Technologies, Santa Clara, CA, USA), with an alternately coupled double pump system. Final separation was performed on an analysis column (Kinetex 2.6 µm EVO-C18, 100 Å, 50 × 2.1 mm; Phenomenex, Torrance, CA, USA). Aldosterone concentration was determined using calibration curves based on the ratio between signals for analyzed samples and an internal standard. 

### 2.5. Statistical Analysis

Group characteristics were reported as mean and standard deviation or median and interquartile range, as appropriate, for continuous variables and as counts and percentages for categorical variables. Group comparison was performed using an independent samples *t*-test for normally distributed variables and a non-parametric test (Mann–Whitney) for non-normally distributed continuous variables. Spearman’s rank correlation coefficients were used to examine relationships among continuous variables. IBM SPSS Statistics for Macintosh, Version 27.0 (IBM Corp, Armonk, NY, USA) was used for statistical analyses. 

## 3. Results

Baseline characteristics and postural hemodynamics are presented in Table 1. Higher heart rate (69 ± 11 bpm vs. 63 ± 11 bpm, *p* = 0.006) and diastolic blood pressure (DBP, 73 ± 9 vs. 69 ± 9 mmHg, *p* = 0.008) were observed in POTS when supine. Likewise, during active standing, both heart rate and DBP were higher in POTS at 1, 3, and 5 min (Table 1). 

**Figure 2:**
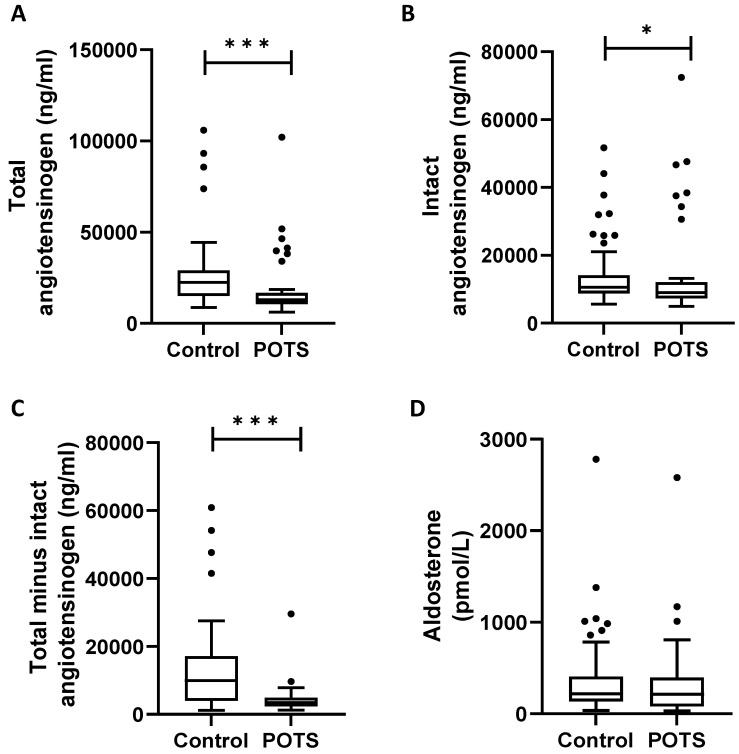
Plasma concentrations of total angiotensinogen (**A**), intact angiotensinogen (**B**), and renin activity (total − intact angiotensinogen, (**C**)) and aldosterone (**D**) in POTS and controls. POTS, postural orthostatic tachycardia syndrome. * *p*-value < 0.05; *** *p*-value < 0.001. “Total minus intact angiotensinogen” indicates “des-angiotensin-I-angiotensinogen” generated 1:1 in relation to angiotensin I, which corresponds to renin activity.

Renin activity, expressed as angiotensin I generation, was decreased in patients with POTS (median, 3406 ng/mL; interquartile range (IQR), 2275–4767) when compared to healthy controls (median, 9949 ng/mL; IQR, 3937–17175; *p* < 0.001 (Figure 2). In contrast, aldosterone concentration did not differ between the groups (median, 218 pmol/L; IQR, 81–397) vs. 218 pmol/L; IQR, 135–407; *p* = 0.26) (Figure 2).

**Figure 3:**
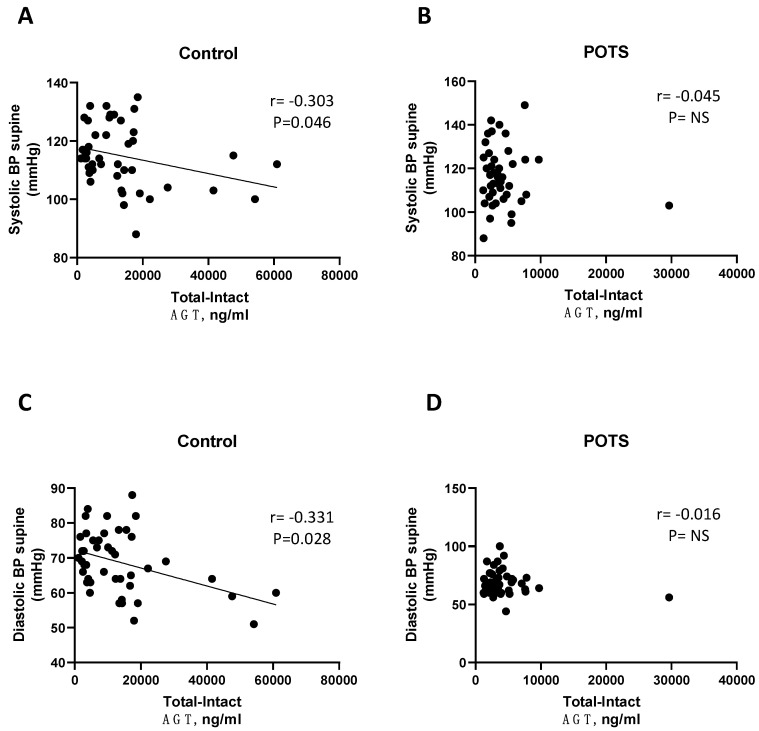
Correlation between supine systolic and diastolic blood pressure and renin activity in controls (**A**,**C**) and POTS (**B**,**D**). AGT, angiotensinogen; BP blood pressure; POTS, postural orthostatic tachycardia syndrome. “Total − Intact AGT” indicates “des-angiotensin-I-AGT” generated 1:1 in relation to angiotensin I, which corresponds to renin activity.

**Figure 4:**
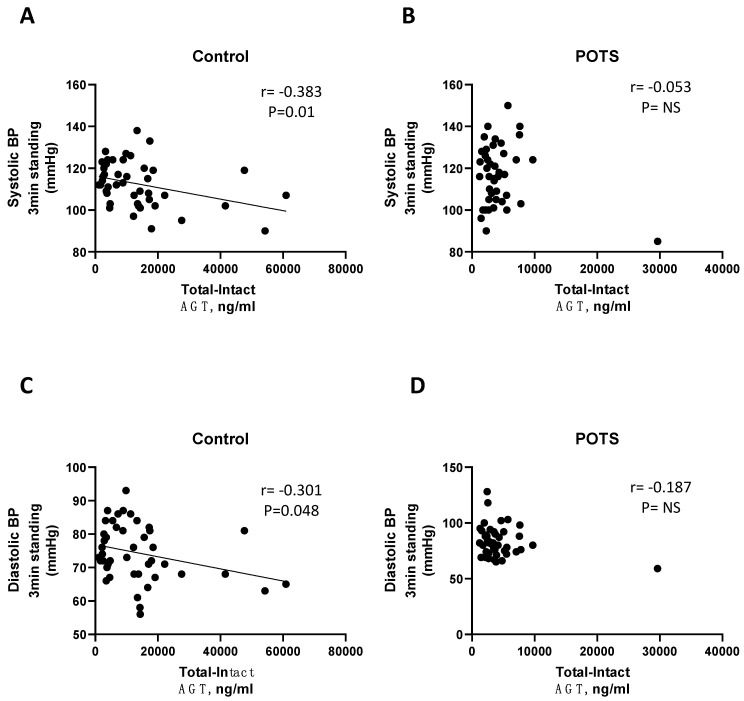
Correlation between systolic/diastolic blood pressure after 3 min of standing and renin activity (total − intact angiotensinogen) in controls (**A**,**C**) and POTS (**B**,**D**). AGT, angiotensinogen; BP blood pressure; POTS, postural orthostatic tachycardia syndrome. “Total − Intact AGT” indicates “des-angiotensin-I-AGT” generated 1:1 in relation to angiotensin I, which corresponds to renin activity.

As shown in Table 2 and Table 3 and Figure 3 and Figure 4, renin activity demonstrated an inverse correlation with both supine and standing blood pressure in controls (*p* < 0.05), but not in subjects with POTS, except for DBP after 5 min standing. A weak correlation was observed between aldosterone and supine heart rate in POTS *(p* = 0.049, Table 4). 

## 4. Discussion 

In this study, we report a downregulation of renin activity in patients with postural orthostatic tachycardia syndrome (POTS) when compared to healthy controls. In contrast, plasma aldosterone levels in controls and POTS did not differ. Moreover, an inverse correlation between renin activity and both supine and orthostatic blood pressure values was observed in healthy controls but was clearly absent in POTS. 

### 4.1. Renin–Angiotensin–Aldosterone System 

The renin–angiotensin–aldosterone system regulates plasma sodium concentration, arterial blood pressure, and extracellular volume [10,11]. The main initiator of this cascade is enzyme renin [23]. When renal blood flow is reduced, juxtamedullary epithelioid cells in the kidneys convert angiotensinogen into angiotensin I by renin secretion. Then, angiotensin-converting enzyme (ACE), released from endothelial cells in the lungs, converts angiotensin I to angiotensin II and degrades active bradykinin, which plays an important role in blood pressure regulation [13,24,25]. Angiotensin II is a peptide hormone which is a potent vasoconstrictor regulating blood pressure by binding to AT1 and AT2 receptors and by stimulating aldosterone release from the adrenal cortex [26]. Aldosterone increases sodium and water uptake in the kidneys and simultaneously excretes potassium [27]. 

### 4.2. Autonomic Nervous System and RAAS

When the autonomic control of the cardiovascular system is intact, the blood pressure increases when upright due to peripheral vasoconstriction as a compensatory response to preserve adequate blood perfusion to the brain. Paradoxically, renin release decreases due to increased perfusion of blood. As observed in this study, renin activity was inversely correlated with BP in the control group, both supine and during active standing, which can be explained by this compensatory mechanism. However, in POTS, this correlation was absent. These results may be explained by disruption in the feedback loop of the renin–angiotensin–aldosterone system resulting in disturbed regulation of renin activity. Alternatively, this may be a consequence of inappropriate activation or blockade of angiotensin II, type I receptor by autoantibodies, as suggested by a recent study [17]. Previous reports of autoimmunity in POTS revealed antibodies against adrenergic and angiotensin II, type I receptors (AT1R) [15,16,17]. Dysregulation in the adrenergic and angiotensin systems may contribute to hemodynamic disturbances, but it is uncertain how these autoantibodies might produce the characteristic cardiovascular alterations in POTS. A recent study demonstrated that the mere presence of autoantibodies targeting cardiovascular G-protein coupled receptors, among other adrenergic and angiotensin II receptors, cannot differentiate POTS patients from controls [28]. A more intricate antibody–receptor interaction was also proposed, as demonstrated by cell-based functional assays [15,16,17]. Hypothetically, if autoantibodies affected function of AT1R independently of angiotensin generation then aldosterone secretion would still be intact despite decreased renin activity. However, this cannot explain the mechanism behind decreased renin activity, which has been found by other workers in this field [13].

### 4.3. Hemodynamic Alterations in POTS

Patients with POTS had a higher supine and standing heart rate and diastolic blood pressure. Increased heart rate is a typical feature of POTS pathophysiology and is explained by different possible mechanisms such as increased sympathetic activity, reflex tachycardia due to hypovolemia, and autoimmunity against adrenergic receptors [1]. Increased diastolic blood pressure, also reported in previous studies [13], is thought to be a consequence of increased baseline vasoconstriction due to increased sympathetic activity. 

### 4.4. Previous Reports on RAAS in POTS

Previous studies have not been unanimous regarding RAAS activity in POTS. Raj et al. reported paradoxically unchanged renin activity and lower aldosterone in patients with POTS despite their lower plasma volume [29]. In contrast, Stewart et al. observed reduced plasma renin activity among POTS patients with lower blood volume but not among those with normal plasma volume [30]. Moreover, increased angiotensin II activity was observed in the subgroup with lower blood volume. In another study based on a relatively larger patient series (165 vs. 66 normal controls), plasma renin activity tended to be higher in patients with POTS, whereas plasma aldosterone was decreased [31]. In our study aldosterone concentration did not differ between the groups and did not correlate with hemodynamic changes when standing either in POTS or in controls. Consequently, the reports on renin activity and aldosterone concentration in POTS patients have been largely inconsistent, which may be related to the heterogeneity of studied patient populations and control groups, as well as different analytical methods. Further verification of our report through independent patient populations and alternative analytical methods is warranted.

## 5. Limitations

This is a single-center study with all the limitations that are thus imposed. All participants were asked to stop cardiovascular medications and salt tablets for 48 h prior to testing, but they were not on a low sodium diet. Increased salt diet, which some POTS patients pursue, may lead to altered hormonal activity in the renin–angiotensin–aldosterone cascade.

Another important aspect is the relatively small cohort size. A larger study sample with independent populations may offer confirmation of these findings. Furthermore, angiotensinogen and aldosterone levels were not sampled at each BP measurement. The sampling occurred once and served as baseline levels. For a more accurate assessment, it would be ideal for blood sampling to occur at 0 (supine), 1, 3, and 5 min (upright). 

## 6. Conclusions

Renin activity, but not aldosterone concentration, is downregulated in patients with POTS. Moreover, renin activity in POTS is dissociated from supine and standing blood pressure levels in contrast to healthy individuals. These findings suggest impaired renin function in POTS, which may direct future therapeutic approaches.

## Figures and Tables

**Table 1:** Baseline characteristics and postural hemodynamic parameters in POTS and controls.

Covariates	POTS + (n = 46)	POTS − (n = 48)	*p*-Value
Age, years	27 ± 9	30 ± 9	0.053
Female sex, n (%)	39 (85)	36 (75)	0.238
SBP, supine (mmHg)	117 ± 13	115 ± 11	0.221
DBP, supine (mmHg)	73 ± 9	69 ± 9	0.008
HR, supine (bpm)	69 ± 11	63 ± 11	0.006
SBP, standing 1 min (mmHg)	121 ± 18	114 ± 11	0.13
DBP, standing 1 min (mmHg)	84 ± 13	75 ± 8	<0.001
HR, standing 1 min (bpm)	93 ± 19	79 ± 13	<0.001
SBP, standing 3 min (mmHg)	117 ± 16	112 ± 14	0.080
DBP, standing 3 min (mmHg)	81 ± 13	72 ± 10	0.001
HR, standing 3 min (bpm)	96 ± 18	83 ± 12	<0.001
SBP, standing 5 min (mmHg)	117 ± 18	112 ± 13	0.08
DBP, standing 5 min (mmHg)	80 ± 15	74 ± 11	0.015
HR, standing 5 min (bpm)	93 ± 18	84 ± 13	0.005

Systolic blood pressure (SBP), Diastolic blood pressure (DBP), Heart rate (HR), Beats per minute (bpm).

**Table 2:** Correlation between baseline RAAS-markers and systolic blood pressure.

Spearman Correlation Coefficient		Systolic BP Supine	Systolic BP Standing 1 min	Systolic BP Standing 3 min	Systolic BP Standing 5 min
		(mmHg)	(mmHg)	(mmHg)	(mmHg)
Control	Angiotensin I (ng/mL)	r = −0.303	r = −0.507	r = −0.383	r = −0.320
	*p*-value	0.046	0.001	0.01	0.039
	Aldosterone (pmol/L)	r = −0.71	r = −0.143	r = −0.062	r = −0.046
	*p*-value	0.648	0.361	0.69	0.775
POTS	Angiotensin I (ng/mL)	r = 0.061	r = −0.157	r = 0.015	r = −0.073
	*p*-value	0.687	0.321	0.925	0.649
	Aldosterone (pmol/L)	r = −0.128	r = −0.222	r = −0.276	r = −0.244
	*p*-value	0.407	0.163	0.073	0.129

BP blood pressure; POTS, Postural orthostatic tachycardia syndrome.

**Table 3:** Correlation between baseline RAAS-markers and diastolic blood pressure.

Spearman Correlation Coefficient		Diastolic BP Supine	Diastolic BP Standing 1 min	Diastolic BP Standing 3 min	Diastolic BP Standing 5 min
		(mmHg)	(mmHg)	(mmHg)	(mmHg)
Control	Angiotensin I (ng/mL)	r = −0.331	r = −0.308	r = −0.300	r = −0.276
	*p*-value	0.028	0.044	0.048	0.077
	Aldosterone (pmol/L)	r = −0.090	r = −0.200	r = −0.142	r = −0.046
	*p*-value	0.059	0.2	0.358	0.772
POTS	Angiotensin I (ng/mL)	r = −0.245	r = −0.051	r = −0.201	r = −0.278
	*p*-value	0.101	0.748	0.191	0.079
	Aldosterone (pmol/L)	r = −0.106	r = −0.244	r = −0.137	r = −0.175
	*p*-value	0.492	0.125	0.382	0.28

BP blood pressure; POTS, Postural orthostatic tachycardia syndrome; RAAS, Renin–Angiotensin–Aldosterone System.

**Table 4:** Correlation between baseline RAAS-markers and heart rate.

Spearman Correlation Coefficient		HR Supine	HR Standing 1 min	HR Standing 3 min	HR Standing 5 min
		(bpm)	(bpm)	(bpm)	(bpm)
Control	Angiotensin I (ng/mL)	r = −0.269	r = 0.054	r = 0.052	r = −0.248
	*p*-value	0.078	0.73	0.739	0.114
	Aldosterone (pmol/L)	r = 0.209	r = 0.035	r = 0.184	r = 0.233
	*p*-value	0.174	0.824	0.232	0.138
POTS	Angiotensin I (ng/mL)	r = −0.068	r = −0.106	r = −0.017	r = −0.195
	*p*-value	0.654	0.504	0.914	0.227
	Aldosterone (pmol/L)	r = 0.299	r = 0.082	r = 0.282	r = 0.150
	*p*-value	0.049	0.611	0.067	0.363

BP blood pressure; POTS, Postural orthostatic tachycardia syndrome; RAAS, Renin–Angiotensin–Aldosterone System.

## Data Availability

The data that support the findings of this study are available from the corresponding author, upon reasonable request.
